# Lipoxin A_4_ Counter-regulates Histamine-stimulated Glycoconjugate Secretion in Conjunctival Goblet Cells

**DOI:** 10.1038/srep36124

**Published:** 2016-11-08

**Authors:** Robin R. Hodges, Dayu Li, Marie A. Shatos, Charles N. Serhan, Darlene A. Dartt

**Affiliations:** 1 Schepens Eye Research Institute/Massachusetts Eye and Ear, Department of Ophthalmology, Harvard Medical School, Boston, MA, 02114, USA; 2 Center for Experimental Therapeutics and Reperfusion Injury, Harvard Institutes of Medicine, Department of Anesthesiology, Perioperative, and Pain Medicine, Brigham and Women’s Hospital and Harvard Medical School, 02115, USA

## Abstract

Conjunctival goblet cells synthesize and secrete mucins which play an important role in protecting the ocular surface. Pro-resolution mediators, such as lipoxin A_4_ (LXA_4_), are produced during inflammation returning the tissue to homeostasis and are also produced in non-inflamed tissues. The purpose of this study was to determine the actions of LXA_4_ on cultured human conjunctival goblet cell mucin secretion and increase in intracellular [Ca^2+^] ([Ca^2+^]_i_) and on histamine-stimulated responses. LXA_4_ increased mucin secretion and [Ca^2+^]_i_, and activated ERK1/2 in human goblet cells. Addition of LXA_4_ before resolvin D1 (RvD1) decreased RvD1 responses though RvD1 did not block LXA_4_ responses. LXA_4_ inhibited histamine-stimulated increases in mucin secretion, [Ca^2+^]_i_, and ERK1/2 activation through activation of β-adrenergic receptor kinase 1. We conclude that conjunctival goblet cells respond to LXA_4_ through the ALX/FPR2 receptor to maintain homeostasis of the ocular surface and regulate histamine responses and could provide a new therapeutic approach for allergic conjunctivitis and dry eye diseases.

Conjunctival goblet cells are specialized cells that span the thickness of the conjunctiva from the ocular surface to the stroma. These cells synthesize and secrete mucins which include the gel forming mucin MUC5AC in humans and in rats^1^,^2^. These mucins are responsible for maintenance of ocular surface hydration, lubrication, and prevention of destructive interaction of foreign bodies and pathogens with the conjunctiva. Goblet cells also play a role in the innate immune response of the conjunctiva and can be activated by cytokines produced during inflammation^3^,^4^.

In the context of the ocular surface, the types of inflammation observed include seasonal allergic conjunctivitis, and dry eye syndrome^5^,^6^. Allergic conjunctivitis alone affects 15–25% of Americans^6^. Dry eye disease is a chronic, multifactorial disease and can be a result of graft vs host disease, autoimmune diseases, normal aging or refractive and cataract surgeries^7^,^8^,^9^,^10^,^11^. It has been estimated that the overall cost of dry eye disease treatment in the United States is more than $3.8 billion though that number is likely underestimated^12^. Uncontrolled inflammation is a hallmark of these ocular surface diseases causing redness, itching, and discomfort and creating a significant impact on quality of life. There are few effective treatments for these diseases and most are only palliative.

During the allergic response, mast cells are recruited to the ocular surface, degranulate, and release histamine and leukotrienes (LT)^13^,^14^. We previously showed that goblet cells of the conjunctiva play an active role in the response of the ocular surface to histamine and leukotriene challenge^15^,^16^,^17^. All four receptors (H1-H4) for histamine as well as cysteinyl leukotriene receptors, CysLT_1_ and CysLT_2_, are expressed in goblet cells^17^. Activation of each of the these receptor subtypes caused an increase in intracellular [Ca^2+^] ([Ca^2+^]_i_) and high molecular weight glycoconjugate secretion including MUC5AC^15^,^17^.

Termination of inflammation occurs with the biosynthesis of the specialized pro-resolution mediators (SPMs) resolvins (Rvs), lipoxins (LX), maresins, and protectins from omega-3 and -6 essential fatty acids^18^. These resolution-phase mediators alter the magnitude and the duration of the inflammatory response through mechanisms involving counter regulation of inflammatory mediators as well as phagocytosis of microbes and cell debris^18^. Recent evidence suggests that LXs and Rvs also play a role under normal, physiological conditions^19^,^20^,^21^,^22^,^23^. In conjunctival goblet cells, RvD1 and its epimer aspirin-triggered RvD1 (AT-RvD1), and RvE1 appear to have two functions (1) alone these compounds increase [Ca^2+^]_i_, activate extracellular regulated kinase (ERK) 1/2, and stimulate mucin secretion and (2) block LTD_4_- and histamine-stimulated increase in [Ca^2+^]_i_ and mucin secretion^19^,^20^.

LXA_4_ and lipoxin B_4_ (LXB_4_) are both biosynthesized from arachidonic acid. LXA_4_ binds to the ALX/FPR2 receptor causing a conformational change leading to stimulation of pro-resolution pathways^24^. Similar to RvD1 and AT-RvD1, LXA_4,_ LXB_4_, and several stable analogs of LXA_4_ alone increased [Ca^2+^]_i_ in conjunctival goblet cells from rats. LXA_4_ also increased mucin secretion utilizing the signaling pathways of phospholipase C, -D, and A_2_^19^. The increase in [Ca^2+^]_i_ stimulated by LXA_4_ was directly linked to mucin secretion as chelation of intracellular Ca^2+^ blocked secretion^19^. In the present study, we investigated the actions of LXA_4_ with cultured human conjunctival goblet cells, as well as the impact of LXA_4_ on histamine-stimulated increase in [Ca^2+^]_i_, mucin secretion, and ERK 1/2 activation in rat and human goblet cells. In human goblet cells, LXA_4_ binds to the ALX/FPR2 and GPR32 receptors while RvD1 binds to GPR32 receptors. In rat, LXA_4_ and RvD1 preferentially bind to the ALX/FPR2 receptor. In addition, we report that LXA_4_ utilizes β-adrenergic receptor kinase (βARK) 1 to counter-regulate the H1 histamine receptor.

## Results

### Action of LXA_4_ on [Ca^2+^]_i_ and Protein Secretion in Human Goblet Cells

**Figure 1:**
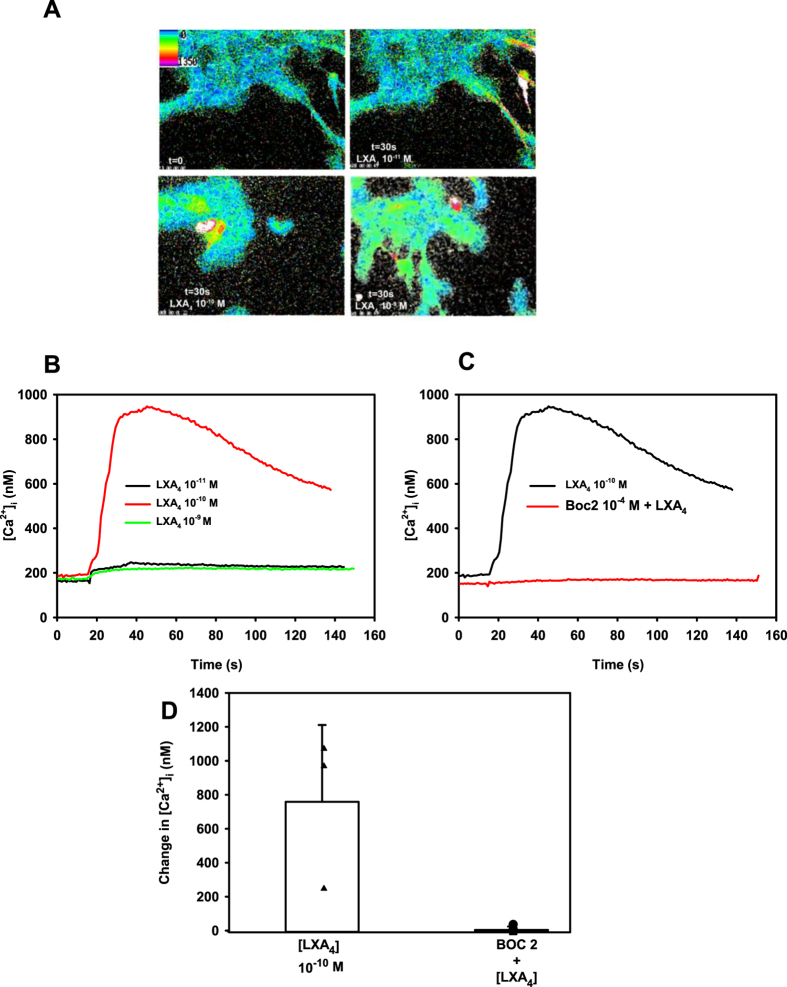
Lipoxin A_4_ (LXA_4_) Increases Intracellular [Ca^2+^] ([Ca^2+^]_i_) in Human Conjunctival Goblet Cells. Pseudo-color images of goblet cells stimulated with increasing concentrations of LXA_4_ (10^−11^–10^−9^ M) is shown in (**A**). Effect of LXA_4_ (black line, LXA_4_ 10^−11^ M; red line, LXA_4_ 10^−10^ M; green line, LXA_4_ 10^−9^ M) on [Ca^2+^]_i_ over time is shown in (**B**) Goblet cells were preincubated with BOC-2 (10^−4^ M) for 30 min prior to addition of LXA_4._ and [Ca^2+^]_i_ was measured. Effect over time with LXA_4_ 10^−10^ M is shown in (**C**) Change in peak [Ca^2+^]_i_ was determined (closed triangles) and is shown in (**D**). Change in peak [Ca^2+^]_i_ was calculated and is shown in (**D**). Data are either the mean of the response over time from 3 individuals (**B**) or individual values or mean ± SD from the same 3 individuals (**D**).

We previously demonstrated that LXA_4_ stimulated an increase in [Ca^2+^]_i_ and mucin secretion in rat goblet cells^19^. To investigate the actions of LXA_4_ on goblet cells grown from human conjunctiva, cells were stimulated with LXA_4_ from 10^−11^ M–10^−9^ M and the increase in [Ca^2+^]_i_ was measured. Pseudo colored images of goblet cells treated with increasing concentrations of LXA_4_ are shown in Fig. 1A. LXA_4_ increased [Ca^2+^]_i_ in a concentration dependent manner with increases in peak [Ca^2+^]_i_ of 97.6 ± 31.2, 762.0 ± 259.4, and 50.0 ± 14.4 nM at 10^−11^, 10^−10^, and 10^−9^ M, respectively (Fig. 1B,D). The increase at 10^−10^ M LXA_4_ was increased above basal (p = 0.03).

LXA_4_ has been shown to bind to the ALX/FPR2 receptor in goblet cells isolated from rat conjunctiva^19^. To determine whether LXA_4_ acts via ALX/FPR2 receptors in human goblet cells, goblet cells were pretreated with the ALX/FPR2 antagonist BOC-2 (10^−4^ M) for 30 min, prior to addition of LXA_4_ (10^−10^ M). Similar to rat goblet cells, in cultured human cells BOC-2 inhibited LXA_4_-stimulated increase in [Ca^2+^]_i_ at 10^−10^ M by 98.5 ± 1.1% to 13.3 ± 11.7 nM (p=0.05) (Fig. 1C,D).

**Figure 2:**
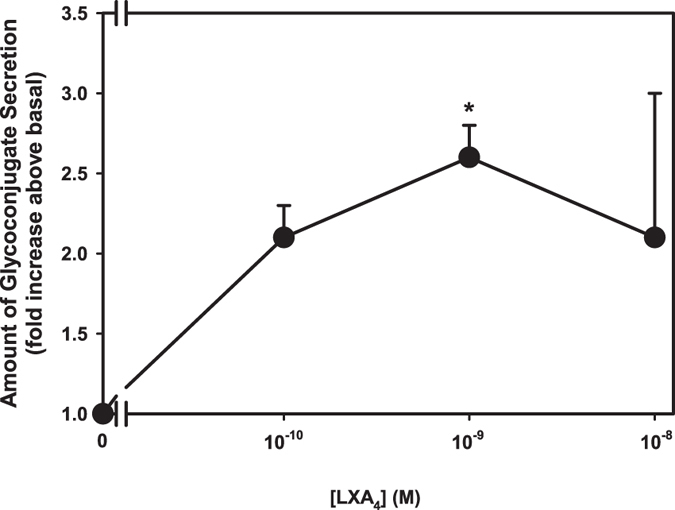
Lipoxin A_4_ (LXA_4_) Stimulates Glycoconjugate in Human Conjunctival Goblet Cells. Goblet cells were stimulated with LXA_4_ (10^−10^–10^−8^ M) for 2 h and glycoconjugate secretion measured by ELLA. Data are mean ± SD from 3 individuals. *indicates significance difference from basal.

Next, the actions of LXA_4_ on glycoprotein secretion from cultured human goblet cells was determined. Goblet cells were serum-starved for 2 h and LXA_4_ was added (10^−10^–10^−8^ M) for 2 h and glycoconjugate secretion measured. LXA_4_ (10^−9^ M) increased secretion 2.6 ± 0.1 fold above basal (p = 0.01, Fig. 2). In cells from the same individuals, histamine, as a positive control, increased glycoconjugate secretion 2.5 ± 0.3 fold above basal (p = 0.005, data not shown). These data show that in human goblet cells, similar to rat goblet cells, LXA_4_ activates the ALX/FPR2 receptor to increase [Ca^2+^]_i_ and stimulate glycoconjugate secretion.

### Presence and Localization of ALX/FPR2 Receptors in Human Conjunctival Goblet Cells

**Figure 3:**
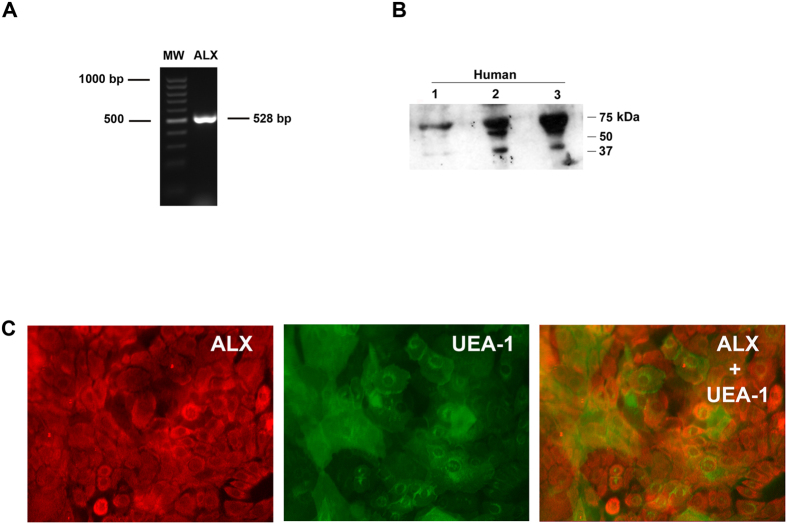
ALX/FPR2 Receptor is Present in Human Conjunctival Goblet Cells. RNA was isolated from cultured goblet cells, and RT-PCR performed with primers to human ALX/FPR2 receptor and is shown in (**A**). ALX/FPR2 was also detected by Western blot analysis in human conjunctival goblet cells (**B**). Each lane in B represents a different individual. ALX/FPR2 was also detected by immunofluorescent techniques (**C**). ALX/FPR2 is shown in red (left micrograph) while UEA-1, which binds to L-fucose containing high moleculuar weight glycoproteins including mucins in goblet cells, is shown in green (middle micrograph). Overlay of ALX/FPR2 and UEA-1 is shown in right micrograph. Micrographs are representative of results from 3 individuals.

As LXA_4_ stimulates an increase in [Ca^2+^]_i_ and glycoconjugate secretion and the ALX/FPR2 receptor inhibitor, BOC-2, blocks LXA_4_-stimulated increase in [Ca^2+^]_i_, we confirmed that ALX/FPR2 is expressed in human goblet cells. RT-PCR, using primers specific for this receptor, was performed. As shown in Fig. 3A, one band at the expected size was detected. The receptor is also expressed at the protein level as detected by western blot analysis from cells grown from 3 individuals (Fig. 3B). The ALX/FPR2 receptor is known to be glycosylated which could account for the multiple bands observed^24^. In cultured human goblet cells, ALX/FPR2 (shown in red) was present throughout the cytosol of the cells (Fig. 3C). UEA-1, shown in green was used to confirm the identity of cultured goblet cells (Fig. 3C). There was substantial overlap in the localization of ALX/FPR2 and UEA-1. These data confirm that ALX/FPR2 is present in human goblet cells.

### Interaction of LXA_4_ and RvD1 via ALX/FPR2 and GPR32

**Figure 4:**
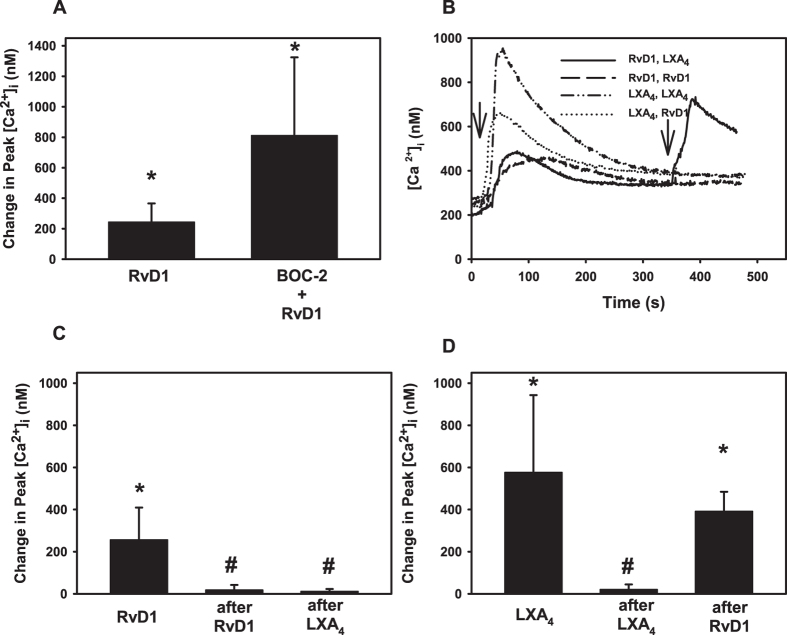
Receptor Interaction of Resolvin D1 (RvD1) and Lipoxin A_4_ (LXA_4_) in Human Conjunctival Goblet Cells. Human goblet cells were preincubated with BOC-2 (10^−4^ M) or vehicle for 30 min prior to addition of RvD1 and [Ca^2+^]_i_ was measured. Change in peak [Ca^2+^]_i_ was calculated and shown in (**A**). RvD1 was added alone or 5 min after addition of RvD1 or LXA_4_ (**B**,**C**) or LXA_4_ was added alone or 5 min after addition of LXA_4_ or RvD1 (**B**,**D**) and [Ca^2+^]_i_ measured. Panel (**B)** is mean of [Ca^2+^]_i_ over time while (**C**,**D**) are mean ±SD in the change in peak [Ca^2+^]_i_ from 3 individuals. Data from (**B–D**) are from the same individuals. Arrows indicate addition of either RvD1 or LXA_4_. *indicates significance difference from basal; ^#^indicates significance difference from RvD1 or LXA_4_.

The ALX/FPR2 receptor has multiple agonists which can bind to it. These agonists include RvD1, the protein annexin A1, as well as LXA_4_^25^. In addition to ALX/FPR2, RvD1 and LXA_4_ also bind to the receptor GPR32^26^, which we previously demonstrated to be present in cultured human goblet cells^20^. We explored the interaction between LXA_4_ and RvD1 with ALX/FPR2 and GPR32 in human goblet cells. In a first set of experiments, we determined the extent to which RvD1 binds to ALX/FPR2 in human goblet cells. Goblet cells were preincubated with ALX/FPR2 inhibitor BOC-2 (10^−4^ M) prior to stimulation with RvD1 (10^−8^ M) and [Ca^2+^]_i_ was measured. In the absence of inhibitor, RvD1 increased [Ca^2+^]_i_ by 242.8 ± 70.8 nM (Fig. 4A). Preincubation with BOC-2 had no effect on the RvD1 response (p = 0.14).

In a second set of experiments, the following experimental paradigm was used: addition of first agonist was followed 5 minutes later by addition of second agonist and the increase in peak [Ca^2+^]_i_. was measured approximately 30 seconds after addition of agonist. In cultured human goblet cells, the addition of RvD1 (10^−8^ M) first caused a peak increase in [Ca^2+^]_i_ of 256.2 ± 62.5 nM (Fig. 4B,C). A second addition of RvD1, 5 minutes after the first, resulted in a peak increase of [Ca^2+^]_i_ of 17.9 ± 13.8 nM (Fig. 4B,C). This was a decrease from the response obtained when RvD1 was added first (p = 0.04). If LXA_4_ is added first and RvD1 is added 5 min later, the RvD1 response was 11.3 ± 11.3 nM. This is also a decrease from the response obtained if RvD1 is added first (p = 0.03).

If LXA_4_ (10^−9^ M) is added first, the initial response obtained was a change in peak [Ca^2+^]_i_ of 576.5 ± 149.8 nM (Fig. 4B,D). A second addition of LXA_4_ resulted in a decrease from the first peak and was 20.4 ± 13.8 nM (p = 0.04). If RvD1 is given first, the addition of LXA_4_ resulted in change in peak [Ca^2+^]_i_ of 391.7 ± 53.7 nM. This was not different from the result obtained with LXA_4_ added first (p = 0.4, Fig. 4B,D) though it is increased from basal (p = 0.002). The data shown in Fig. 4C,D are from the same individuals.

These data indicate that in human goblet cells, RvD1 preferentially binds to GPR32 which desensitizes the receptor to a second addition of RvD1 while the LXA_4_ response is not altered. In contrast, LXA_4_ binds to both the ALX/FPR2 and GPR32 receptor and a second addition of LXA_4_ desensitizes both receptors to a subsequent addition of either LXA_4_ or RvD1.

### LXA_4_ Inhibits Histamine-stimulated Increase in Glycoconjugate Secretion, [Ca^2+^]_i_, and ERK Activation

**Figure 5:**
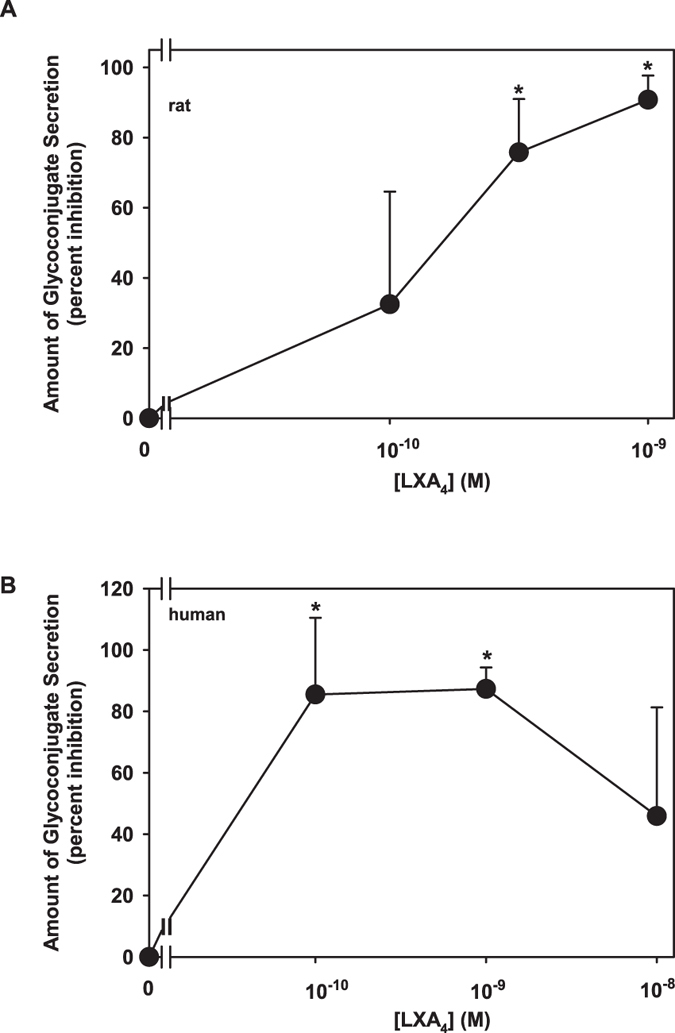
LXA_4_ Blocks Histamine-stimulated Glycoconjugate Secretion from Rat and Human Goblet Cells. Goblet cells from rat (**A**) or human (**B**) were preincubated with LXA_4_ (10^−10^–10^−8^ M) for 30 min prior to addition of histamine (His, 10^−5^ M) for 2 h and glycoconjugate secretion measured by ELLA. Data are mean ± SD from 3 rats or 3 individuals. *indicates significance difference from histamine alone.

To examine the effects of LXA_4_ on histamine-stimulated glycoconjugate mucin secretion, rat goblet cells were pretreated with LXA_4_ (10^−10^–10^−9^ M) for 30 min and stimulated with histamine (10^−5^ M) for 2 h. In untreated rat cells, histamine increased mucin secretion 1.9 ± 0.1 fold increase above basal (p=0.002). LXA_4_ blocked histamine-stimulated secretion by 75.8 ± 8.8 and 90.8 ± 4.0% at 3 × 10^−10^ and 10^−9^ M, respectively (p = 0.001 and 0.00002, Fig. 5A). In human goblet cells, histamine increased mucin secretion 2.5 ± 0.3 fold increase above basal (p=0.005). Preincubation with LXA_4_ 10^−10^ and 10^−9^ M blocked histamine-stimulated secretion by 85.5 ± 14.5 and 87.3 ± 8.4%, respectively (p = 0.004 and 0.0003, Fig. 5B).

**Figure 6:**
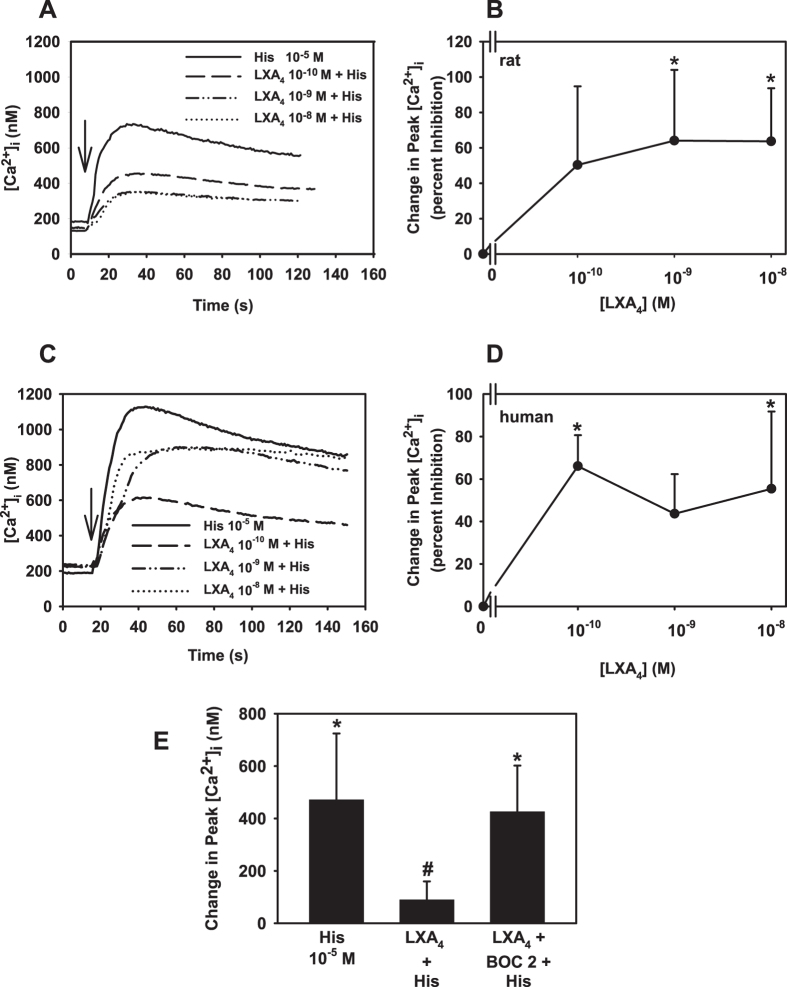
LXA_4_ Uses ALX/FPR2 to Block Histamine-stimulated Increase in [Ca^2+^]_i_ in Rat and Human Goblet Cells. Goblet cells from rat (**A**,**B**) or human (**C**,**D**) were preincubated with LXA_4_ (10^−10^–10^−8^ M) for 30 min prior to addition of histamine (His, 10^−5^ M). Panels A and C are mean of [Ca^2+^]_i_ over time. Arrows indicate addition of histamine. Panels B,D are mean ± SD in the change in peak [Ca^2+^]_i_ from 8 rats or 4 humans. Rat goblet cells were stimulated with either histamine (His, 10^−5^ M); LXA_4_ (10^−9^ M) for 30 min prior to addition of histamine; or preincubated with ALX/FPR2 inhibitor BOC-2 (10^−4^ M) 15 min prior to addition of LXA_4_ (10^−9^ M) which was added 30 min before histamine. The change in peak [Ca^2+^]_i_ was measured and is shown in (**E)**. Data are mean ± SD from 4 rats. *Indicates significance difference from histamine alone. ^#^Indicates significance difference from histamine.

We previously established that histamine increases [Ca^2+^]_i_ in a concentration-dependent manner which was blocked by RvD1 and AT-RvD1^20^. To determine if LXA_4_ also blocks histamine responses, cultured goblet cells were preincubated with LXA_4_ prior to stimulation with histamine. In goblet cells cultured from rat, histamine (10^−5^ M) increased [Ca^2+^]_i_. with a peak of 587.1 ± 92.3 nM (p = 0.0002, Fig. 6A). Preincubation with LXA_4_ (10^−10^–10^−8^ M) decreased histamine-stimulated increase in [Ca^2+^]_i_ at 10^−9^ and 10^−8^ M with maximum inhibition occurring at 10^−9^ M LXA_4_ which decreased the histamine response by 64.1 ± 14.1% to 185.6 ± 52.1 nM (p = 0.01, Fig. 6A,B).

Similar results were obtained with cultured human goblet cells. Histamine (10^−5^ M) stimulated an increase in peak [Ca^2+^]_i_ 1152.5 ± 173.9 nM (p=0.006, Fig. 6C,D). Preincubation with LXA_4_ (10^−10^–10^−8^ M) decreased histamine-stimulated increase in [Ca^2+^]_i_ at all concentrations with maximum inhibition occurring at 10^−10^ M LXA_4_ which decreased the histamine response by 66.1 ± 7.3% to 378.6 ± 92.6 nM (p = 0.005, Fig. 6C,D).

To ensure that the actions of LXA_4_ on histamine response are mediated by the ALX/FPR2 receptor, rat goblet cells were pretreated with BOC-2 (10^−4^ M) for 15 min prior to addition of LXA_4_ (10^−9^ M) for 30 min. The [Ca^2+^]_i_ was then measured in response to histamine (10^−5^ M). In the absence of BOC-2 and LXA_4_, the change in peak [Ca^2+^]_i_ in response to histamine was 586.8 ± 173.1 nM (p = 0.01, Fig. 6E). LXA_4_ added first reduced the histamine response by 90.3 ± 1.9% to 55.5 ± 8.5 nM (p = 0.03). Preincubation with BOC-2 reversed the inhibition by LXA_4_ on the histamine response and increased [Ca^2+^]_i_ by 495.0 ± 76.6 nM (Fig. 6E).

**Figure 7:**
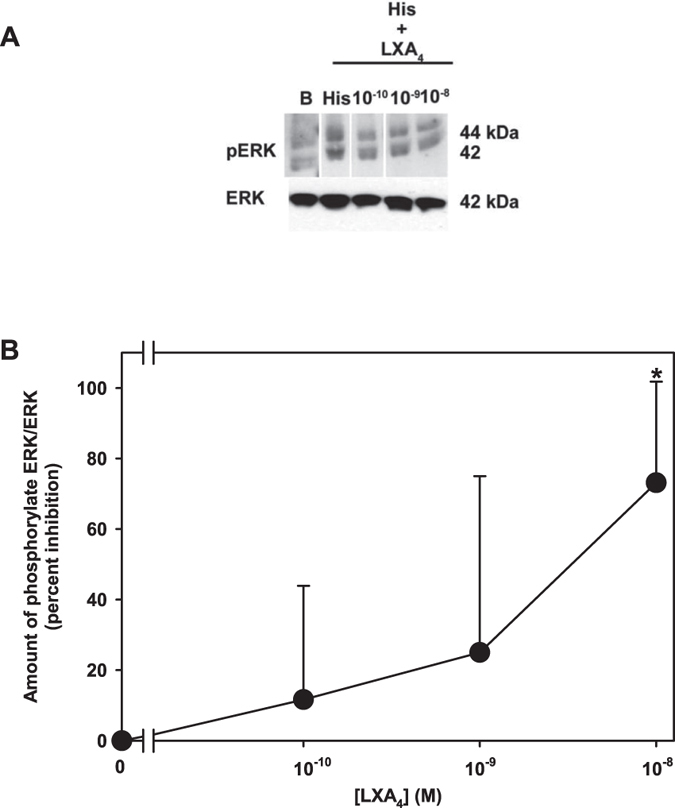
LXA_4_ Blocks Histamine-stimulated ERK 1/2 Activation in Rat Goblet Cells. Rat goblet cells were preincubated with LXA_4_ (10^−10^–10^−8^ M) for 30 min prior to addition of histamine (His, 10^−6^ M) for 5 min and amount of activated (phosphorylated) and total ERK determined by Western blot analysis. Representative blot is shown in (**A**). Upper blot has been rearranged for ease of comparison. Mean ± SD from 4 rats are shown in (**B**) *indicates significant difference from histamine alone.

Histamine activates ERK 1/2 to stimulate glycoconjugate secretion which was also blocked by RvD1 and AT-RvD1^15^,^20^. To determine if LXA_4_ also blocks histamine-stimulated ERK 1/2 activity, rat goblet cells were preincubated with LXA_4_ (10^−10^–10^−8^ M) for 30 min prior to incubation with histamine (10^−6^ M) for 5 min and ERK 1/2 activity was measured. Histamine increased ERK 1/2 activity 1.3 ± 0.1 fold increase above basal (p=0.04, Fig. 7A,B). LXA_4_ decreased this response at all concentrations (Fig. 7A). When four independent experiments were analyzed, inhibition with LXA_4_ 10^−8^ M decreased histamine response by 64.4 ± 19.4% to 1.1 ± 0.1 fold increase above basal (p = 0.002, Fig. 7B).

### ALX/FPR2 Uses βARK1, but Not Protein Kinase C, to Block the H1 Histamine Receptor Simulated Increase in [Ca^2+^]_i_

**Figure 8:**
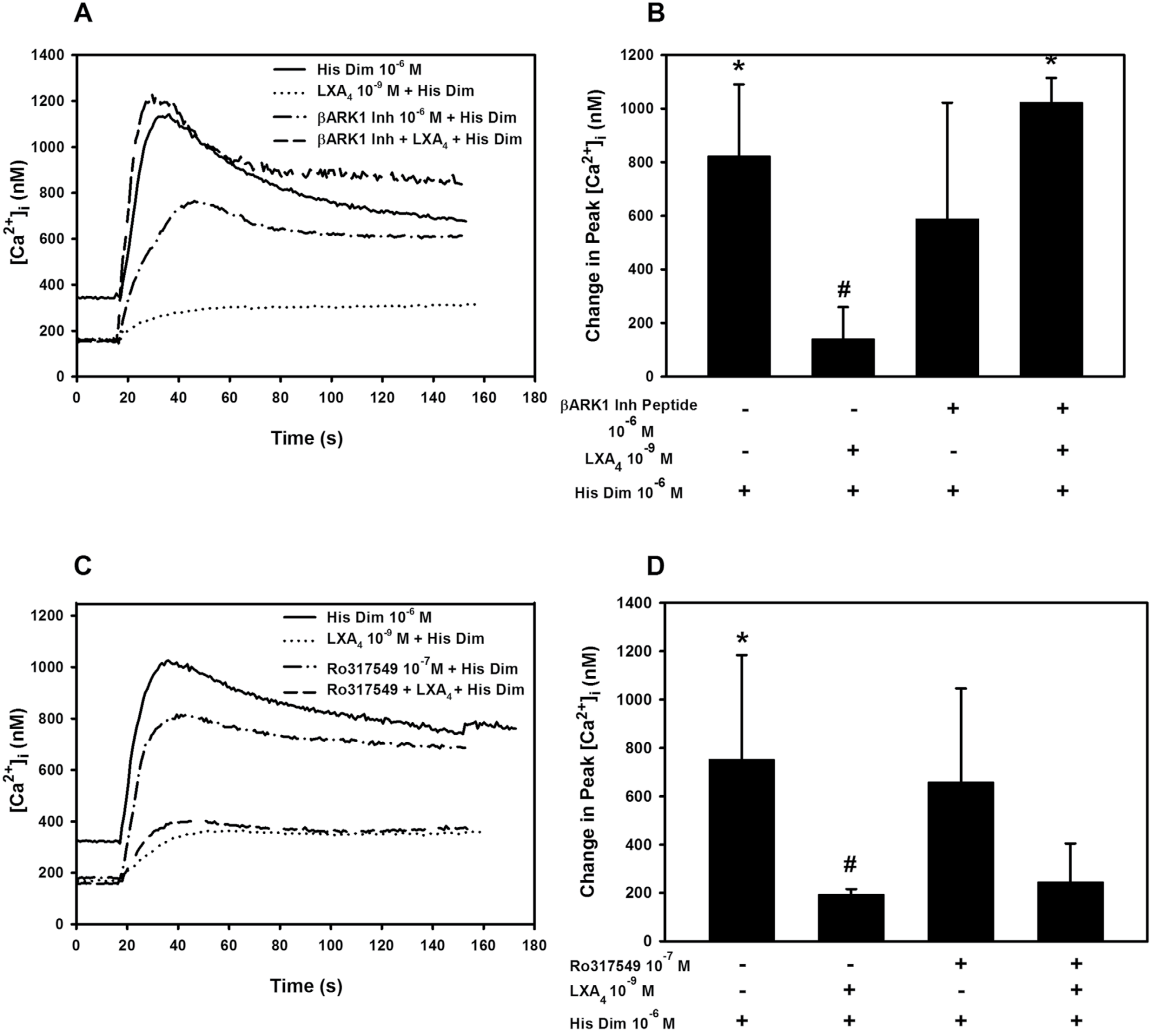
LXA_4_ Uses β-Adrenergic Receptor Kinase 1 to Block H1 Histamine Receptor. Rat goblet cells from rat were stimulated with either histamine dimaleate (His Dim, 10^−6^ M); preincubated with LXA_4_ (10^−9^ M) for 30 min prior to addition of the His Dim; or preincubated with β-adrenergic receptor kinase 1 inhibitor peptide (βARK 1 Inh peptide, 10^−6^ M, (**A,B**) or with the PKC inhibitor Ro317549 (10^−7^ M, (**C,D**) added 30 min before His Dim; or preincubated with β-adrenergic receptor kinase 1 inhibitor peptide (βARK 1 Inh peptide, 10^−6^ M, (**A,B**) or with the PKC inhibitor Ro317549 (10^−7^ M, (**C,D**) for 15 min prior to addition of LXA_4_ that was added 30 min before His Dim. Change in [Ca^2^]_i_ over time is shown in (**A**,**C**) and change in peak [Ca^2+^]_i_ is shown in (**B**,**D**). Data are mean ± SD from 3 (**A,B**) or 4 (**C,D**) individuals. *indicates significance difference from basal; ^#^indicates significance difference from His Dim alone.

Examination of the H1 histamine receptor for phosphorylation sites using Scan Site (http://scansite.mit.edu/), showed that this receptor has consensus sequences for β-adrenergic receptor kinase 1 (βARK1), also known as G-protein coupled receptor kinase (GRK)-2 and protein kinase C (PKC). We previously demonstrated that RvD1 binding to GPR32 activates both these kinases to counter-regulate the H1 histamine receptor to block the increase in [Ca^2+^]_i_^20^. To determine if ALX/FPR2 and LXA_4_ also use βARK1 and/or PKC to counter regulate histamine H1 receptor, rat goblet cells were pretreated with either LXA_4_ or LXA_4_ plus inhibitors to βARK1 and PKC. The increase in [Ca^2+^]_i_ in response to the specific H1 receptor agonist, histamine dimaleate was measured in cultured rat goblet cells. Pretreatment with LXA_4_ decreased the histamine dimaleate stimulated increase in [Ca^2+^]_i_ from 823.7 ± 154.1 nM above basal in the absence of LXA_4_ to 140.9 ± 68.7 nM (p = 0.02, Fig. 8A,B). βARK1 inhibitor peptide (10^−6^ M) alone did not have an effect of the histamine dimaleate (p = 0.16, Fig. 8A,B). When cells were pretreated with βARK1 inhibitor peptide followed by LXA_4_, blockage of the histamine dimaleate response by LXA_4_ was completely reversed (Fig. 8A,B).

To investigate the role of PKC in counter-regulation of H1 receptor by LXA_4_, rat goblet cells were preincubated with the PKC inhibitor Ro317549 (10^−7^ M). In these experiments, histamine dimaleate increased [Ca^2+^]_i_ by 751.7 (p=0.01, ± 216.1 nM (Fig. 8C,D). This response was decreased by LXA_4_ and was 193.3 ± 11.3 nM (p = 0.04, Fig. 8C,D). Ro317549 alone had no effect on histamine dimaleate response (Fig. 8C,D). Addition of the PKC inhibitor prior to LXA_4_ had no effect on the LXA_4_ blockage of histamine dimaleate (Fig. 8C,D).

These data indicate that the activation of the ALX/FPR2 uses βARK1 but not PKC to counter regulate the H1 histamine receptor in rat goblet cells. This is in contrast to RvD1 that uses both βARK1 and PKC to counter regulate the H1 receptor.

## Discussion

**Figure 9:**
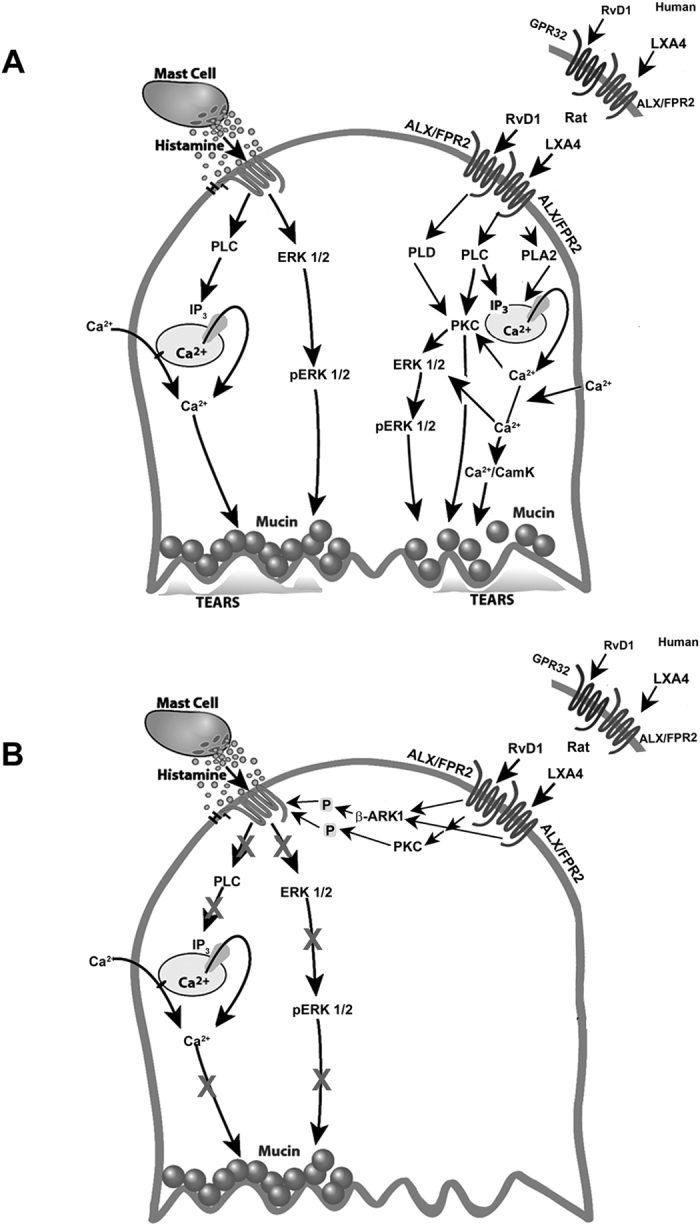
Schematic Diagram of Pathways Activated by LXA_4_ in Rat and Human Conjunctival Goblet Cells to Stimulate Mucin Secretion and Inhibit Histamine-stimulated Mucin Secretion. In rat goblet cells, histamine via the H1 histamine receptor subtype activates phospholipase (PL) –C to stimulate extracellular-regulated kinase 1/2 (ERK 1/2) which leads to mucin secretion. In addition, inositol trisphosphate (IP_3_) is produced which also leads to release of Ca^2+^_i_ and activation of Ca^2+^ channels leading to mucin secretion Also in rat goblet cells activation of the ALX/FPR2 receptor stimulates PLC, -D, and A2. These phospholipases activate ERK 1/2 through phosphorylation (pERK 1/2), and protein kinase C (PKC). IP_3_ is produced which leads to release of Ca^2+^_i_ and activation of Ca^2+^ channels leading to mucin secretion. (**A**) Activation of ALX/FPR2 by either LXA_4_ or RvD1 activates β-adrenergic receptor kinase 1 (βARK1) to counter-regulate the H1 histamine receptor to prevent histamine-stimulated mucin secretion (**B**). In human goblet cells, RvD1 binds to GPR32 receptor and regulates goblet cells (**A,B**). The rat homolog of the human GPR32 if present remains to be identified.

Our results demonstrate that LXA_4_ plays a role in goblet cell function in both normal non-inflamed conditions and acute inflammatory conditions. Our hypothesis is that the ALX/FPR2 receptor is present in human conjunctival goblet cells and activation of the receptor by LXA_4_ stimulates an increase in [Ca^2+^]_i_ and mucin secretion, which in rat goblet cells is protective in the eye and involves activation of phospholipase (PL) C, PLD, and PLA2 signaling pathways (Fig. 9A)^19^. In circumstances such as inflammation or pharmacological addition, LXA_4_ inhibits histamine-stimulated increase in [Ca^2+^]_i_, ERK 1/2 activation, and mucin secretion through the counter-regulation of histamine receptor by βARK1 (Fig. 9B). This may be relevant in controlling excessive histamine release into the conjunctiva.

It is currently not known if any cells in the conjunctiva, including goblet cells, produce and secrete LXA_4_. Along these lines, Gronert *et al*. have demonstrated that the epithelial cells of the cornea endogenously express LXA_4_ and the amount is increased upon wounding^22^. This LXA_4_ could then diffuse via the tears to the goblet cells to stimulate mucin secretion.

While LXA_4_ is an appreciated pro-resolution mediator, the results from several studies indicate that LXA_4_ and other pro-resolution mediators can also play a role within other organs in, physiological conditions that maybe organ specific. For example, LXA_4_ is endogenously produced in the cornea and lacrimal gland under non-inflamed conditions^17^. RvD1 and AT-RvD1, similar to LXA_4_, alone stimulate conjunctival goblet cell functions^20^. These results imply that these mediators could assist in the maintenance of the normal homeostasis of the ocular surface by regulating goblet cell mucin secretion that is linked to ocular surface health.

Allergic conjunctivitis is the most common type of inflammation of the ocular surface. In this condition, histamine interacts with H1-H4 histamine receptors, all of which are expressed in rat and human conjunctival goblet cells^15^. Histamine also increases [Ca^2+^]_i_ and mucin secretion in a concentration dependent manner^15^. Pre-incubation with LXA_4_ blocked histamine-stimulated increase in [Ca^2+^]_i_, mucin secretion and ERK 1/2. Thus, LXA_4_ likely acts as a pro-resolution mediator acting on goblet cells of the conjunctiva to return mucin levels to normal. LXA_4_ is likely to have similar effects on histamine-stimulated responses in other tissues. For example, LXA_4_ inhibits histamine release from human lung mast cells^27^ and histamine-stimulated paw edema in mice^28^.

This study examined the actions of LXA_4_ on conjunctival goblet cells only. The ocular surface consists of multiple cell types and is covered by tears, which are a complex film that overspreads the ocular surface^29^. The actions of LXA_4_ have not been tested on other types of cells on the ocular surface nor in the presence of tears.

Cultured human goblet cells often react similarly to LXA_4_ as cultured rat goblet cells. In goblet cells from both species, LXA_4_ stimulated an increase [Ca^2+^]_i_, and mucin secretion to the same extent (current study and^20^). Mucin secretion stimulated by cysteinyl leukotrienes in human goblet cells was also similar to that obtained with rat goblet cells^17^. There does appear to be several differences between rat and human goblet cells. In human goblet cells, the concentration LXA_4_ required to maximally inhibit histamine-stimulated increase in [Ca^2+^]_i_ was 10 fold less that than that required in rat goblet cells. An additional difference was demonstrated by experiments involving interactions of LXA_4_ and RvD1 with their receptors. In rat goblet cells, initial addition of either LXA_4_ or RvD1 blocked the increase in [Ca^2+^]_i_ stimulated by a second addition of either LXA_4_ or RvD1 indicating that these two SPMs bind to the same receptor^19^. However in human goblet cells, while an initial addition of LXA_4_ blocks the RvD1 response, an initial addition of RvD1 does not block the LXA_4_ response. In addition, BOC-2 does not alter RvD1-stimulated increase in [Ca^2+^]_i_. These results support the notion that in human cells RvD1 preferentially activates GPR32 while LXA_4_ activates both receptors (Fig. 9). LXA_4_ is an established agonist of ALX/FPR2 and has been shown to bind to GPR32 in human phagocytes^26^. It is also known that RvD1 binds to both ALX/FPR2 and GPR32^26^. At this point it is not known if a rat homolog of GPR32 is present and functional in rat goblet cells. Since GPR32 has not yet been identified in rat, it is possible that RvD1 only binds to ALX/FPR2 in these rat cells. There are many other situations in which rat differs from human including regulatory T cell phenotypes^30^, wound healing in skin^31^, and glomerulonephritis^32^.

We previously showed the mechanism by which RvD1 prevents the actions of histamine in rat goblet cells^20^. We found that RvD1 counter-regulates the H1 histamine receptor by activation of both βARK1 and PKC to prevent the H1 specific agonist-stimulated increase in [Ca^2+^]_i_^20^. In contrast to RvD1, only an inhibitor of βARK1 reversed the LXA_4_ inhibition of H1 histamine receptor. Cooray *et al*. have demonstrated that ALX/FPR2 receptor can form hetero- and homodimers depending on the agonist bound^24^. Thus RvD1 and LXA_4_ could form different dimer formations in rat goblet cells.

The signaling pathways activated by LXA_4_ after binding to ALX/FPR2 are dependent on the cell type (Table 1). LXA_4_ acting through ALX/FPR2 stimulated an increase in [Ca^2+^]_i_, chemotaxis and adherence in human monocytes^33^,^34^. In human neutrophils, ALX/FPR2 activation leads to lipid remodeling, arachidonic acid release, and activation of phospholipase D (PLD) via PKC with a small increase in [Ca^2+^]_i_^35^. LXA_4_ had no effect on [Ca^2+^]_i_^36^ in human astrocytoma cells while it increased [Ca^2+^]_i_, in human bronchial epithelia as well as increased the number of tight junctions and Cl^−^ secretion and decreased Na^+^ absorption^37^,^38^,^39^. Thus LXA_4_ can have variable effects on [Ca^2+^]_i_ and other cell functions and cellular responses to LXA_4_ need to be determined for each cell type.

In conclusion, we demonstrate that ALX/FPR2 receptors are present on cultured human goblet cells, and that LXA_4_ alone increases [Ca^2+^]_i_, mucin secretion and ERK 1/2 activation. In addition, LXA_4_ counter-regulates the H1 histamine receptor to block its activation thereby returning the ocular surface to homeostasis. LXA_4_ thus plays a critical role in ocular surface health and maintenance in physiological conditions. In addition, LXA_4_ protects the ocular surface from challenges of the external environment that induce ocular surface inflammatory and allergic diseases. Thus LXA_4_ and this receptor axis may provide the basis for new therapeutic treatments for these diseases.

## Materials and Methods

Synthetic LXA_4_ was purchased from EMD Millipore (Billerica, MA) and RvD1 was purchased from Cayman Chemical, Ann Arbor, MI). Both compounds were dissolved in ethanol as supplied by the manufacturer and were stored at −80 °C with minimal exposure to light. Immediately prior to use, the SPMs were diluted in with Krebs-Ringer bicarbonate buffer with HEPES (KRB-HEPES, 119 mM NaCl, 4.8 mM KCl, 1.0 mM CaCl_2_, 1.2 mM MgSO_4_, 1.2 mM KH_2_PO_4_, 25 mM NaHCO_3_, 10 mM HEPES, and 5.5 mM glucose [pH 7.45]) to the desired concentrations and added to the cells. The cells were then incubated at 37 °C in the dark. Daily working stock dilutions were discarded following each experiment. N-BOC-Phe-Leu-Phe-Leu-Phe (BOC-2) was purchased from Genescript (Piscataway, NJ).

### Human Tissue

Human conjunctiva was obtained from Eversight (Ann Arbor, MI). Tissue was placed in Optisol media within 18 h after death.

### Animals

Male Sprague-Dawley rats (Taconic Farms, Germantown, NY) weighing between 125 and 150 g were anesthetized with CO2 for 1 min, decapitated, and the bulbar and forniceal conjunctival membranes removed from both eyes. All experiments were approved by the Schepens Eye Research Institute Animal Care and Use Committee and carried out in accordance to the protocols approved by this committee.

### Cell Culture

Goblet cells from human and rat conjunctiva were grown in organ culture as described and extensively characterized previously^4^,^16^,^17^,^40^,^41^,^42^. The tissue plug was removed after nodules of cells were observed. First passage goblet cells were used in all experiments. The identity of cultured cells was periodically checked by evaluating staining with antibody to cytokeratin 7 (detects goblet cell bodies) and the lectin Ulex europaeus agglutinin (UEA)-1 (detects goblet cell secretory product) to ensure that goblet cells predominated.

### Measurement of [Ca^2+^]_i_

Goblet cells were incubated for 1 h at 37 °C with KRB-HEPES with 0.5% BSA containing 0.5 μM fura-2/AM (Invitrogen, Grand Island, NY), 8 μM pluronic acid F127, and 250 μM sulfinpyrazone followed by washing in KRB-HEPES containing sulfinpyrazone. Inhibitors were added for the last 30 min of the fura-2 incubation. Calcium measurements were made with a ratio imaging system (InCyt Im2; Intracellular Imaging, Cincinnati, OH) using wavelengths of 340 and 380 nm and an emission wavelength of 505 nm. At least 10 cells were selected in each experimental condition. Data were collected in real time and are presented as the actual [Ca^2+^]_i_ with time or as the change in peak [Ca^2+^]_i_. Change in peak [Ca^2+^]_i_ was calculated by subtracting the average of the basal value (no added agonist) from the peak [Ca^2+^]_i_. Although data are not shown, the plateau [Ca^2+^]_i_ was affected similarly to the peak [Ca^2+^]_i_.

### Measurement of Glycoconjugate Secretion

Cultured goblet cells were serum starved for 2 h before use and then stimulated with either LXA_4_ or histamine in serum-free RPMI 1640 supplemented with 0.5% BSA for 2 h. Inhibitors were added 30 min prior to stimulation. Goblet cell secretion was measured using an enzyme-linked lectin assay (ELLA) with the lectin UEA-I. UEA-1 detects high molecular weight glycoconjugates containing L-fucose including mucin MUC5AC produced by goblet cells^43^. The media were collected and analyzed for the amount of lectin-detectable glycoconjugates, which quantifies the amount of goblet cell secretion as described earlier^17^. Glycoconjugate secretion was expressed as fold increase over basal that was set to 1.

### Reverse Transcriptase (RT)-PCR

Cultured human goblet cells were homogenized in TRIzol and total RNA was isolated. One microgram of purified total RNA was used for complementary DNA (cDNA) synthesis using the Superscript First-Strand Synthesis system for RT-PCR (Invitrogen, Carlsbad,CA). The cDNA was amplified by the polymerase chain reaction (PCR) using primers specific to human ALX/FPR2 receptor using the Jumpstart REDTaq Readymix Reaction Mix (Sigma-Aldrich, St. Louis, MO) in a thermal cycler (Master Cycler, Eppendorf, Hauppauge, NY). The primers were from published sequences^44^. The forward primer sequence was GGA TTT GCA CCC ACT GCA TTT and reverse primer was ATC CAA GGT CCG AGA TCA C. These primers generated a product of 528 base pairs. β−Actin served as the positive control. The primers were from published sequences^45^. The conditions were as follows: 5 min at 95 °C followed by 35 cycles of 1 min at 94 °C, 3 s at annealing temperature for 1 min at 72 °C with a final hold at 72 °C for 10 min. Samples with no cDNA served as the negative control. Amplification products were separated by electrophoresis on a 1.5% agarose gel and visualized by ethidium bromide staining.

### Western blotting analyses

Cultured goblet cells were homogenized in RIPA buffer (10 mM Tris-HCl [pH 7.4], 150 mM NaCl, 1% deoxycholic acid, 1% Triton X-100, 0.1% SDS, and 1 mM EDTA) containing a protease inhibitor cocktail (Sigma-Aldrich, St. Louis, MO). The lysate was centrifuged at 2000 g for 30 min at 4 °C. Proteins were separated by sodium dodecyl sulfate-polyacrylamide gel electrophoresis and processed for western blotting. The antibody against the ALX/FPR2 receptor (Novus Biologics, Littleton, CO) was diluted 1:1000. To measure activation of ERK 1/2, LXA_4_ was added 30 min prior to histamine (10^−6^ M) for 5 min. The antibody against phosphorylated (active) ERK 1/2 was used diluted 1:200 and total ERK 1/2 (Santa Cruz Biotechnologies, Santa Cruz, CA) was diluted 1:500. Immunoreactive bands were visualized by the enhanced chemiluminescence method. The films were analyzed with Image J software (http://rsbweb.nih.gov/ij/). Values for phosphorylated ERK 1/2 were normalized to total ERK 1/2. Control value was set as 1.

### Immunofluorescence Microscopy

First passage cells were grown on glass cover slips and were fixed in 4% formaldehyde diluted in phosphate buffered saline (PBS, 145 mM NaCl, 7.3 mM Na_2_HPO_4_, and 2.7 mM NaH_2_PO_4_ (pH 7.2)) for 4 hours at 4 °C. The coverslips were rinsed for 5 minutes in PBS, and nonspecific sites were blocked by incubation with 1% bovine serum albumin, and 0.2% Triton X-100 in PBS for 45 minutes at room temperature. ALX/FPR2 receptor antibody (Novus Biologics) was used at 1:100 dilution overnight at 4 °C. UEA-1 directly conjugated to FITC (Sigma-Aldrich, St. Louis, MO) was used at a dilution of 1:300 to identify goblet cells. Secondary antibodies were conjugated to Cy 3 (Jackson ImmunoResearch Laboratories, West Grove, PA) was used at a dilution of 1:150 for 1 h at room temperature. Negative control experiments included incubation with the isotype control antibody. The cells were viewed by fluorescence microscopy (Eclipse E80i; Nikon, Tokyo, Japan) and micrographs were taken with a digital camera (Spot; Diagnostic Instruments, Inc, Sterling Heights, MI).

### Statistical analysis

Results were expressed as the fold-increase above basal. Results are presented as mean ± SD. Data were analyzed by ANOVA followed by post-hoc Tukey or Student’s *t*-test. P < 0.05 was considered statistically significant.

## Additional Information

**How to cite this article**: Hodges, R. R. *et al*. Lipoxin A_4_ Counter-regulates Histamine-stimulated Glycoconjugate Secretion in Conjunctival Goblet Cells. *Sci. Rep.* **6**, 36124; doi: 10.1038/srep36124 (2016).

**Publisher’s note**: Springer Nature remains neutral with regard to jurisdictional claims in published maps and institutional affiliations.

## Figures and Tables

**Table 1:** LXA_4_ via ALX/FPR2 Signaling in Human Cell Types.

Tissue	Actions of LXA_4_	References
Human monocytes; THP1	↑[Ca^2+^]_i_ chemotaxis, adherence	33,34
Human neutrophils	↑PKC to PLD; AA release; small increase in [Ca^2+^]_i_; ↓chemotaxis; ↓apoptosis delay	24,35,46
Human astrocytoma cells	↓IL-β induced IL-8 and ICAM-1; no effect on [Ca^2+^]_i_	36
Human bronchial epithelia	↑Tight junctions, [Ca^2+^]_i_, Cl^-^ secretion and ALX translocation, air/surface height; ↓ENaC activity	38

PKC- protein kinase C; PLD – phospholipase D; AA – arachidonic acid; IL-1β - interleukin 1β; IL-8 – interleukin 8; ICAM-1 - intercellular adhesion molecules; EnaC- epithelial sodium channel.
